# Mycophenolic Acid Overcomes Imatinib and Nilotinib Resistance of Chronic Myeloid Leukemia Cells by Apoptosis or a Senescent-Like Cell Cycle Arrest

**DOI:** 10.1155/2012/861301

**Published:** 2012-02-23

**Authors:** Claire Drullion, Valérie Lagarde, Romain Gioia, Patrick Legembre, Muriel Priault, Bruno Cardinaud, Eric Lippert, François-Xavier Mahon, Jean-Max Pasquet

**Affiliations:** ^1^Laboratoire Hématopoïèse Leucémique et Cibles Thérapeutiques, INSERM U1035, Université Bordeaux Ségalen, 146 Rue Léo Saignat Bat TP 4e étage, 33076 Bordeaux, France; ^2^IRSET, EA 4427 SERAIC, Université Rennes-1, 2 Avenue du Professeur Léon Bernard, 35043 Rennes, France; ^3^IBGC, UMR CNRS 5095, Université Bordeaux Ségalen, 1 Rue Camille Saint Saëns, 33077 Bordeaux, France

## Abstract

We used K562 cells sensitive or generated resistant to imatinib or nilotinib to investigate their response to mycophenolic acid (MPA). MPA induced DNA damage leading to cell death with a minor contribution of apoptosis, as revealed by annexin V labeling (up to 25%). In contrast, cell cycle arrest and positive staining for senescence-associated **β**-galactosidase activity were detected for a large cell population (80%). MPA-induced cell death was potentialized by the inhibition of autophagy and this is associated to the upregulation of apoptosis. In contrast, senescence was neither decreased nor abrogated in autophagy deficient K562 cells. Primary CD34 cells from CML patients sensitive or resistant to imatinib or nilotinib respond to MPA although apoptosis is mainly detected. These results show that MPA is an interesting tool to overcome resistance in vitro and in vivo mainly in the evolved phase of the disease.

## 1. Introduction

Chronic myeloid leukaemia (CML) is a myeloproliferative disorder characterized by a reciprocal translocation leading to the Philadelphia chromosome (Ph^+^) with a fusion gene BCR-ABL, the molecular hallmark of CML and Ph-positive acute lymphoblastic leukaemia (LAL) [1–3]. The resulting chimeric protein contains the kinase domain of the tyrosine kinase Abl N-terminal fused to a portion of Bcr including its dimerization domain [1]. The constitutive dimerization of Bcr-Abl results in the deregulated activation of the tyrosine-kinase driving uncontrolled proliferation and suppression of apoptosis in the affected hematopoietic cells. This pathophysiology explains the remarkable efficacy of Abl tyrosine kinase inhibitors (TKI) in controlling CML. Indeed, when exposed to TKI, Bcr-Abl expressing cells undergo apoptosis [4]. Although TKIs have represented a tremendous progress in the management of CML patients, resistances to TKI treatment have emerged. About a third of these resistances can be explained by the acquisition of additional mutations in the kinase domain of Abl. These mutations typically impede the inhibitor binding to its target, and second- generation inhibitors have been designed to overcome these resistances whenever possible. In the remaining resistant patients, the mechanisms are certainly more varied and often remain elusive. In an attempt to characterize and so to overcome resistance to TKI, we have generated K562-derived cell lines resistant to imatinib or nilotinib [5, 6]. We and other have shown that amplification of Bcr-Abl, overexpression of stress proteins, or deregulation of Src kinases are among the mechanisms explaining resistance to imatinib and nilotinib [6–9].

We previously reported that treatment with mycophenolic acid (MPA) could induce CML cell death independently of the sensitivity to TKI [10]. MPA, an active metabolite of Mycophenolate mofetil, is a noncompetitive reversible inhibitor of IMPDH (Inosine monophosphate dehydrogenase; EC1.1.1.205) widely used as an immunosuppressive drug. MPA reduces the GTP pool resulting in a cycle arrest mainly in G_0_/G_1_ phase although some blockage in S phase is also reported [11, 12]. It has been reported to act in synergy with imatinib or methotrexate in Bcr-Abl-positive K562 cells [13, 14]. MPA is described as a potent necrosis inducer on activated lymphocytes [15]. Because the level of expression of IMPDH is increased in leukemic cells in comparison to normal cells and MPA is able to induce the death of both TKI sensitive and resistant CML cells, it could be interesting to identify how CML cells died in response to the agent [16, 17]. The interplay between these mechanisms of cell death in killing Bcr-Abl expressing cells has not been thoroughly characterized, especially in the context of resistance to TKI. In this study, we investigated cell death triggered by MPA in Bcr-Abl expressing cells. Using K562 and CML primary cells, we explored how MPA induced cell death and overridden resistance. In our models, MPA- induced cell deaths mainly relied on senescence and apoptosis. In addition, TKI-induced autophagy acts to rescue the cells from apoptosis, but fails to impair senescence-induced cell death. 

## 2. Materials and Methods

### 2.1. Reagents

RPMI 1640 medium, fetal calf serum (FCS), phosphate buffered saline (PBS), trypan blue, mycophenolic acid, chloroquine, propidium iodide, and the antibody against LC3 were from Sigma (St Quentin Fallavier, France). Tyrosine kinase inhibitors Imatinib and Nilotinib were kindly provided by Novartis Pharma (Basle, Switzerland). The broad caspase inhibitors Z-VAD-fmk were purchased from Peptanova (Sandhausen, Germany). The following antibodies: caspase 3 was from Cell Signalling (Danvers, USA); SQSTM1/p62 and Hsp60 were from Santa Cruz (Bergheimer, Germany). Annexin-V-FITC or APC conjugated were from Beckman coulter (Villepinte, France). 

### 2.2. Cell Lines

The human erythroleukemia Bcr-Abl positive human cell lines used in this study: K562 was from ATCC (CCL-243). TKI-resistant cell lines were derived as previously described for imatinib or nilotinib resistance and designated K562-R or K562-RN [5, 6, 17]. Cells were maintained in RPMI 1640 medium (Sigma, R0883) supplemented with 10% fetal calf serum (FCS; GIBCO, 10270), 2 mM L-glutamine (Invitrogen, 25030), and 100 U/mL penicillin/0.1 mg/mL streptomycin (Invitrogen, 15140) at 37°C in a humidified atmosphere containing 5% CO_2_. Aliquots were taken at 24 h intervals for assessment of cell viability by trypan blue exclusion.

### 2.3. Western Blot

Protein lysates were prepared according to Mahon et al. [6]. Protein concentration was measured by the BCA Protein Assay (Pierce, Rockford IL, USA) and the lysates were stored at −80°C. Equal amounts of protein were separated by electrophoresis on an SDS-PAGE 12.5 or 15% and transferred to a pvdf membrane as described [17]. After blocking, the membrane was incubated with primary antibodies and secondary antibodies. Protein-antibody complexes were detected by an enhanced chemiluminescence immunoblotting ECL (Perkin Elmer, Courtaboeuf, France).

### 2.4. Flow Cytometry

Cells (10^5^ cells) were incubated for 15 min in 500 *μ*L of PBS with 2 mM Ca^2+^, 2 *μ*L of Annexin V-FITC, and 0.25 *μ*g of propidium iodide before flow cytometry analysis on Facscalibur. At least ten thousand events are acquired for statistical analysis.

### 2.5. Cell Cycle

Cells (10^6^ cells) were harvested and washed once with PBS-SVF 5% then permeabilized with PBS 1% PFA 0.1% saponin for 30 min. Cell incubated with PBS- propidium iodide (PI, 0.5 *μ*g/mL containing RNAse) for 15 min before analysis by flow cytometry. Each cell cycle step was quantified by counting cellular events in SubG_1_, G_0_/G_1_, S, and G_2_.

### 2.6. Autophagy Inhibition by RNA Silencing

To stably inhibit autophagy, HIV-1 lentivirus-based vectors were used to introduce shRNAs against ATG7 into cells as previously described [18]. Briefly, shRNAs were cloned in FG12 lentivector, HEK293FT cells were used as packaging cells, and virus production was performed as previously described [19]. The human ATG7 siRNA sequence was 5′-AGG ATA CAG CTG GAG TCA G-3′. Negative-control shRNA was used as already described [20]. To confirm autophagy inhibition by silencing of ATG7, transduced cells were grown in nutrient deprived medium (HBSS) in the absence or in the presence of Bafilomycin A and both LC3B I and II form were detected by Western blot.

### 2.7. SA-*β*-Galactosidase Labeling

Cells (10^6^ cells) were washed with PBS once, before being fixed 4 mins with 3% PFA (paraformaldehyde) then washed once with PBS. Cells were then incubated in a 96-well plate with a mix (1 vol/20 vol) of solution I containing X-Gal (20 mg/mL X-Gal in dimethylformaldehyde, Promega, V3941) and solution II (5 mM ferricyanure, 5 mM ferrocyanure, 2 mM MgCl_2_ 150 mM NaCl, 30 mM Citric acid/phosphate pH = 6) for 24 hours at 37°C [21]. Cells were then washed with PBS and SA-*β*-gal activity was observed by detection of a bleu staining with an inverted Nixon Microscope (Eclipse Ti) and analyzed with the Nikon software NIS. For SA-*β*-gal-positive staining cell quantification, 100 cells were counted on three separate fields and the mean of blue stained cells was calculated as followed (number of blue cells/(number of total cells)). Results are expressed as the percent of SA-*β*-gal-positive cells.

### 2.8. CD34 Cells Isolation and Culture

Mononuclear cells were isolated from blood by Ficoll gradient. CD34 positive cells were purified according to the manufacturer's instructions (Miltenyi Biotech, Germany) and purity was analyzed by flow cytometry using phycoerythrin-conjugated anti-CD34 antibody (Becton Dickinson, France). CD34 cells (10^5^/mL) were grown in X-vivo 10 medium supplemented with 10% fetal calf serum (FCS; GIBCO, 10270), 2 mM L-glutamine (Invitrogen, 25030), and 100 U/mL penicillin/0.1 mg/mL streptomycin (Invitrogen, 15140) at 37°C in a humidified atmosphere containing 5% CO_2_. Cells were incubated with vehicle only, imatinib 1 *μ*M, or MPA 3 *μ*g/mL for 3 days.

### 2.9. Statistical Analysis

Wilcoxon or Friedman tests were used to calculate differences between means; differences were considered significant only when *P* ≤ 0.05.

## 3. Results

### 3.1. MPA Inhibits Proliferation of TKI Sensitive or Resistant K562 Cells by Blocking the Cell Cycle

**Figure 1:**
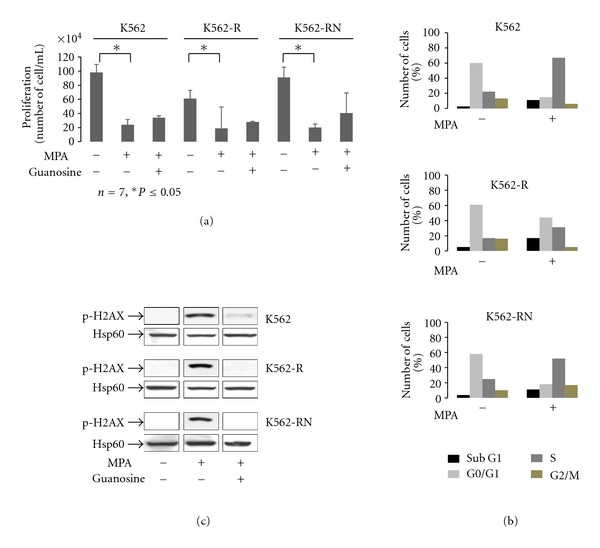
Inhibition of K562 cell proliferation by MPA. K562-S, K562-R and K562-RN cells (2.10^5^/mL) were grown in the presence of vehicle only, MPA (3 *μ*g/mL) or incubated with guanosine (200 *μ*M) and MPA (3 *μ*g/mL) for 3 days. At day 3, cell proliferation was measured by counting cells using trypan blue exclusion assay. Cell counts from triplicate counting are expressed as the mean of 7 independent experiments (a). cells treated as described above were fixed, permeabilized then incubated with propidium iodide (PI 2 *μ*g/mL) and analyzed by flow cytometry for cell cycle measurement. Results are from one experiment representative of five and are expressed as the percent of cells in each phase of cell cycle as described in methods (b). MPA-induced phosphorylation of *γ*H2AX was detected by Western blot on samples as described in (a) (c). Hsp60 was used as loading control.

K562 cells sensitive to imatinib (K562), resistant to imatinib by overexpression of Hsp70 (K562-R) or resistant to nilotinib by overexpression of Lyn kinase (K562-RN) were treated with various doses of MPA (1 to 10 *μ*g/mL). All showed similar sensitivity to the drug as assessed by trypan blue exclusion assay. The dose of 3 *μ*g/mL (9 *μ*M) was chosen for further studies as equally potent in inducing cell death after two to three days in all cells (CML cell lines and primary CD34 cells) and in the range of the plasmatic concentration of MPA-treated patients which is in the range of 1 to 4 *μ*g/mL. MPA inhibited cell proliferation of all three cell lines (Figure 1(a)). Since MPA inhibits IMPDH thus impairing GTP synthesis, addition of exogenous guanosine is expected to rescue the effects of MPA. Addition of guanosine indeed rescued K562 cells from mortality (supplementary Figure 1), but had a modest effect on global proliferation even at longer time. Cell cycle analyses showed that MPA-induced cell death was correlated to a blockage in the S phase with a weak sub-G1 population in all three lines (Figure 1(b)). Consistently, phosphorylation of histone *γ*H2AX, a marker of DNA damage, was induced in all three cell lines, an effect that was prevented by addition of guanosine (Figure 1(c)). 

### 3.2. MPA Induces Apoptosis in K562 Cells

**Figure 2:**
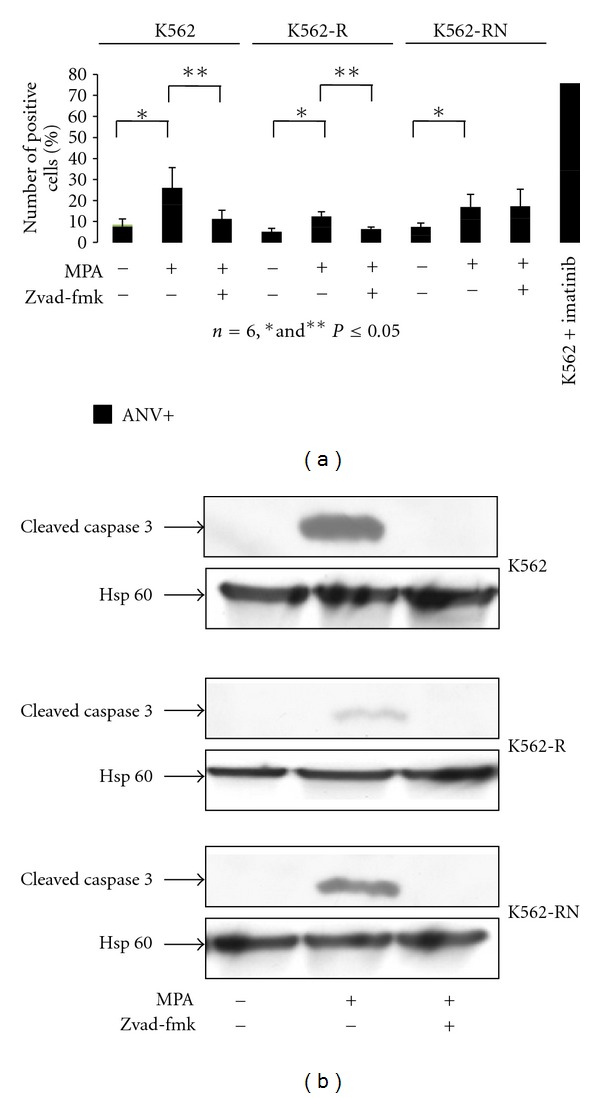
MPA induced a moderated apoptosis. K562-S, K562-R, and K562-RN cells (2.10^5^/mL) were grown in the presence of vehicle only, MPA (3 *μ*g/mL) or incubated with Z-vad-fmk (50 *μ*M) and MPA (3 *μ*g/mL) for 3 days. An aliquot was incubated for 15 min in the presence of annexin-V and propidium iodide. Samples were analyzed by flow cytometry and labelled cells were analyzed as described in Section 2. Results from 6 experiments are expressed as the % of annexin V-labelled cells in comparison to a positive control corresponding to imatinib-treated K562 cells ((a), *n* = 6). Samples treated as above were used for detection of the cleaved form of caspase 3 by Western blot and Hsp60 (as loading control) (b).

In an attempt to determine how MPA-treated cells died, we first look at apoptosis. Annexin V/Propidium Iodide (PI) labelling revealed a weak induction of apoptosis in response to MPA (Figure 2(a)). Even after 3 days of exposure, the number of annexin V-positive cells remained very low compared to the apoptosis induced by imatinib in the TKI-sensitive K562 cell line (Figure 2(a) last lane). Indeed, the weak apoptosis of these cells was further confirmed by the measurement of mitochondrial membrane depolarization using DiOC6 and of PARP cleavage (data not shown). In addition, inhibition of caspase by incubation with 50 *μ*M Z-vad-fmk resulted in a decrease of MPA-induced cleavage of caspase 3 (Figure 2(b)) and in the weak proportion of annexin V-positive cells, except for the K562-RN cell line (Figure 2(a)). These results indicate that MPA induces weak apoptosis in the three K562 cell lines. No significant necrosis (isolated PI stained cell population) was detected.

### 3.3. MPA Induces a Senescent Phenotype in Treated K562 Cells

**Figure 3:**
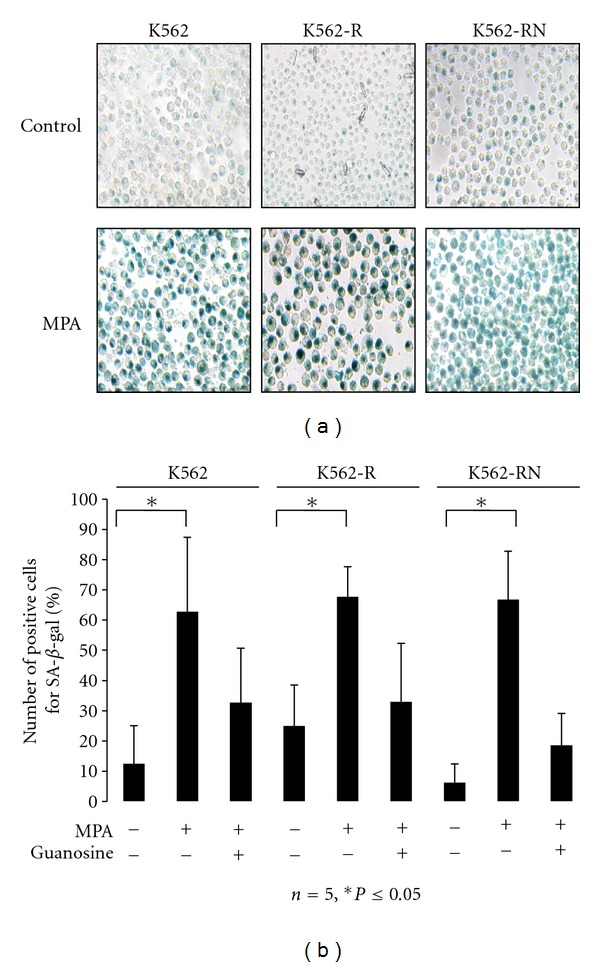
MPA induced SA-*β*-Galactosidase activity. K562-S, K562-R, and K562-RN cells (2.10^5^/mL) were grown in the presence of vehicle only or MPA (3 *μ*g/mL) for 3 days. An aliquot was washed in PBS and 10^5^ cells/well were fixed in PFA and then incubated overnight in a 96-well plate in the presence of X-Gal (1 mg/mL) at 37°C as described in Section 2. The day after, the cells were washed once in PBS and SA-*β*-gal activity was detected by a blue cell staining visualized under an inverted microscope. Pictures were acquired and analyzed using the NIS Nikon software (a). SA-*β*-gal-positive cells were quantified by counting 10^2^ cells on three separate fields for each condition. Results show the mean of five independent experiments (b).

To explain the mortality of our cells in the absence of necrosis and with only limited apoptosis, we studied alternative cell death. Because MPA-treated K562 cells showed an arrest of cell cycle and an increase of cell granularity by flow cytometry, we explored markers of senescence such as an increase of senescence-associated *β*-Galactosidase activity (SA-*β*-gal). The upregulation of p16 and p53, as markers of senescence, cannot be detected in our cells as these genes are deleted in K562 cells. A significant increase of SA-*β*-Gal activity was detected in 70–80% of MPA-treated K562 cells (Figures 3(a) and 3(b)). In addition, MPA induced also foci formation in the nuclei as revealed by DAPI staining (supplementary Figure 2). As was observed with global cell mortality, addition of exogenous guanosine partially abolished the effects of MPA (Figure 3(b)).

### 3.4. Autophagy Limits Apoptosis but Not Senescence in MPA-Treated K562 Cells

**Figure 4:**
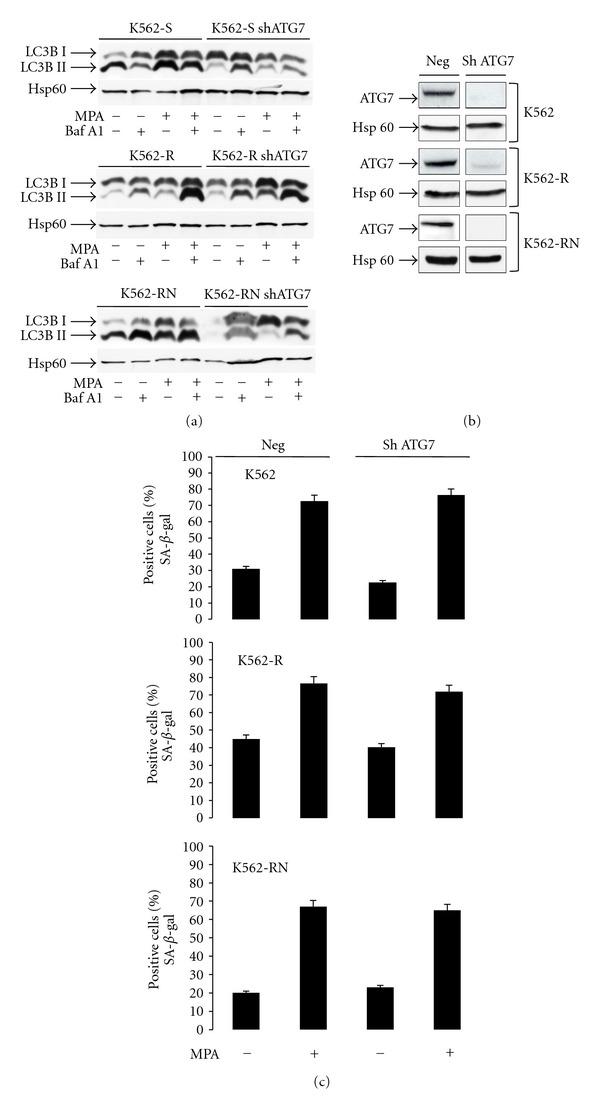
MPA induced autophagy in K562 cells. K562-S, K562-R, and K562-RN cells (2.10^5^/mL) were grown in the presence of vehicle only or MPA (3 *μ*g/mL) for 3 days. Six hours before the end of the incubation, samples were separated in two batches and incubated in the absence or in the presence of bafilomycin A1 (20 nM) to block the autophagic flux. Then, K562 cells were washed once in PBS and lyzed in a modified RIPA buffer for detection by Western blot of LC3B and Hsp60 (as a loading control) (a). K562-S, K562-R and K562-RN cells (2.10^5^/mL) were infected by lentivirus coding for a shRNA anti-ATG7. Lentiviral particles were incubated for 24 h with K562 cells. Then, the cells were washed twice in PBS and grown in the presence of medium for 6 days before sorting based on GFP expression and experimental use. After 3 days, each sample was analysed for ATG7 inhibition by Western blotting (b). K562-S, K562-R, and K562-RN cells (2.10^5^/mL) and K562-S, K562-R, and K562-RN cells (2.10^5^/mL) deficient for ATG7 were grown in the presence of vehicle only, MPA (3 *μ*g/mL). After 3 days, SA-*β*-gal-positive cells were quantified by counting 10^2^ cells on three separate fields for each condition (c). Results show the mean of three experiments.

Because K562 cells sensitive or resistant to imatinib or nilotinib respond to MPA by a senescent-like cell cycle arrest and a weak apoptosis, we next wondered whether autophagy, a mechanism known to modulate various cell death mechanisms, could intervene with MPA-induced K562 cell death. The conversion of the microtubule-associated protein 1 light chain 3 (LC3) from its cytosolic form (LC3BI) to the membrane-bound form (LC3BII) was detected as a bona fide marker of autophagy. Because autophagy is a flux process, MPA- increased LC3BII is visualized by the addition of Bafilomycin A1 (20 nM) 6 hours before the end of MPA incubation. Both forms of LC3 (Figure 4(a)) are upregulated in K562 cells following MPA treatment, indicating that autophagy is actually stimulated in this condition. This was confirmed by SQSTM1/p62 detection by Western blotting (supplementary Figure 3(a)).

In order to understand whether autophagy modulates MPA-induced cell death, we incubated the cells with 3-methyladenine (3-MA) or chloroquine (CQ), two drugs known to inhibit autophagy. MPA-induced apoptosis of K562 cells was significantly increased (2 to 3 fold) when cells were coincubated with 3-MA and MPA or to even higher levels after CQ treatment (supplementary Figures  3(b) and 3(d) available online at Doi:10.1155/2012/861301), indicating that autophagy physiologically limits MPA-induced cell apoptosis. 

We next asked whether autophagy could also alter the MPA-induced senescent-like cell cycle arrest. After treatment of the cells with 3-MA, MPA-induced SA-*β*-gal activity was not significantly modified (supplementary Figure  3(c)), suggesting that inhibition of autophagy had no effect in modulating the senescent response. Chloroquine inhibits the revelation of SA-*β*-gal activity by modifying the pH of lysosomes and we could not use it to emphasize a potential role of autophagy in senescence. As an alternative, we inhibited autophagy by RNA silencing (shRNAs) to shut down the expression of ATG7, a major player of autophagy whose downregulation has been shown to be inhibitory. Inhibition of ATG7 expression was confirmed by Western blotting (Figure 4(b)) and strongly decreased MPA- induced autophagy as reported the decrease of LC3BII level (Figure 4(a)). Even though the expression of ATG7 was completely abolished, no significant difference in MPA-induced SA-*β*-gal activity was observed suggesting that autophagy does not alter MPA induced senescent-like cell cycle arrest (Figure 4(b)).

### 3.5. MPA Overcomes Resistance in Primary CD34 CML Cells

**Figure 5:**
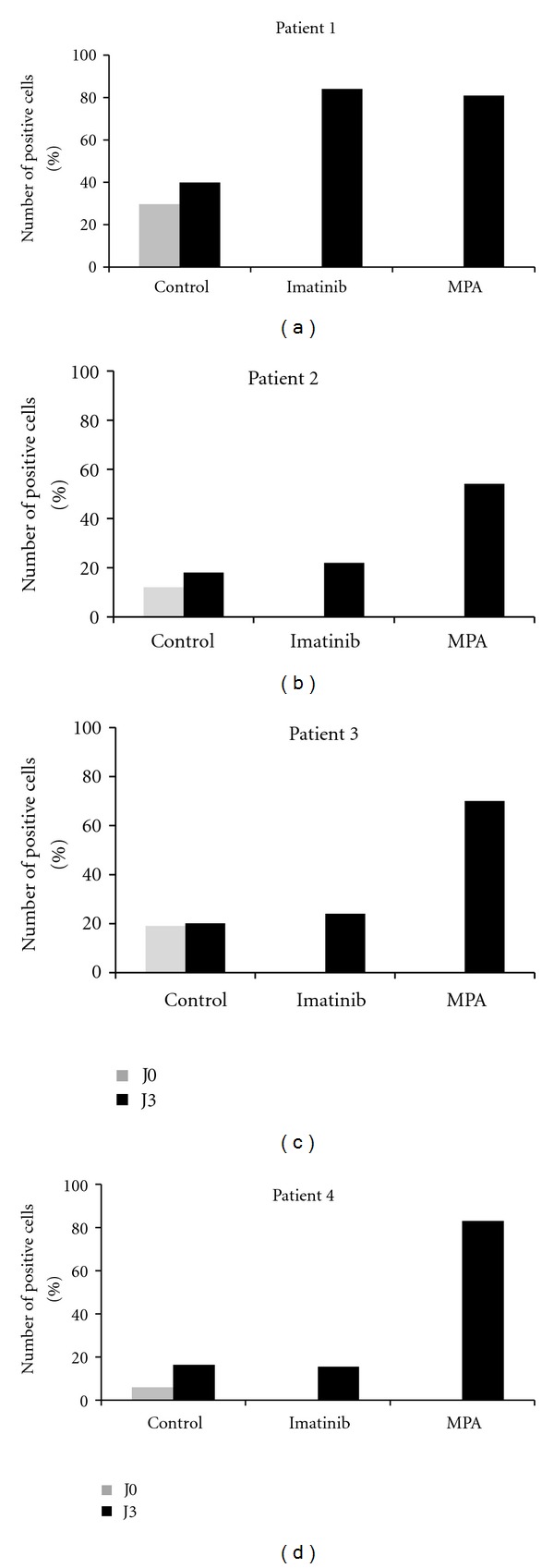
MPA induced apoptosis in CD34 CML cells. Primary CD34 cells isolated from blood samples of CML patients responding to imatinib (patient 1) or resistant to imatinib and nilotinib (patient 2 to 4) were grown in the presence of vehicle only, imatinib 1 *μ*M, or MPA 3 *μ*g/mL for 3 days. Patient 2 and 3 overexpressed several tyrosine kinase such as Src-kinase, whereas patient 4 was mutated in Abl kinase domain harbouring the T315I mutation. Annexin-V binding was detected at day 0 and upon treatment at day 3.

The efficacy of MPA was investigated on CD34 primary cells isolated from blood samples of CML patients responding to imatinib (patient 1) or resistant to imatinib and nilotinib (patient 2 to 4). Patient 2 and 3 overexpressed several tyrosine kinase such as Src-kinase whereas patient 4 was mutated on Abl kinase domain carrying the mutation T315I. All primary cells were grown in the presence of vehicle only, imatinib 1 *μ*M, or MPA 3 *μ*g/mL for 3 days. Annexin-V binding and SA-*β*-Gal was detected at days 0 and upon treatment at day 3. Apoptosis was significantly detected for patient 1 in response to imatinib or MPA. For imatinib- or nilotinib- resistant CML patients only MPA induced apoptosis (Figure 5). When SA-*β*-Gal was revealed, no significant labeling was detected despite an increase in cell size and morphology observed in flow cytometry suggesting that primary cells respond mainly by apoptosis (supplementary Figure 4). Because MPA induced apoptosis of CD34 CML cells, we looked at a possible synergy with imatinib. Interestingly, CD34 cells of TKI sensitive CML patient respond to imatinib by apoptosis but addition of MPA did not increase the apoptotic response (supplementary Figure 5(a)). In contrast, CD34 cells from TKI- resistant patient did not respond to imatinib as suspected but achieved apoptosis in response to MPA. The response of CD34 CML cells to MPA is partially reverted by guanosine addition (supplementary Figure 5(b)) even for the highest MPA concentration required to reach the optimal apoptotic response (supplementary Figure 5(c)).

## 4. Discussion

Despite the major improvement of CML patients' management due to anti-Abl TKIs, one issue remains to be resolved: the emergence of resistances to TKI and relapse [22, 23]. Whatever the mechanisms implicated, they are linked to an impaired TKI-induced apoptosis [24, 25]. This explains that drugs triggering alternative cell death mechanisms are very interesting potential therapeutic tools. MPA is efficiently killing K562 cells, including those resistant to imatinib and nilotinib harbouring different mechanism of resistance. MPA increase mortality of our cells, a specific response since the addition of guanosine prevents it. However, the lack of a total restoration of cell proliferation by guanosine indicates that MPA may also act by other ways although guanosine used at 200 *μ*M may be not enough to refill the GTP pool. Using CD34 CML cells, a similar abrogation effect of MPA was observed with guanosine. A synergy between MPA and imatinib has been reported using CML cell lines which partially overcome resistance to TKI [13]. We did not detect such synergy between imatinib and MPA in CD34 CML cells. It may be because in primary cells, MPA at the concentration used is not able to potentiate imatinib effect. In our hands, MPA alone blocks the cell cycle and induces the death of K562 cells, either sensitive or rendered resistant to TKI. Inhibition of cell cycle by MPA is associated to a blockade to proceed in the S phase. This is similar to the arrest reported by Moosavi et al. using GTP depletion [26]. Apoptosis accounts for only a limited part of MPA-induced K562 death and can be inhibited by preincubation with Z-vad-fmk, a broad inhibitor of caspase. Caspase3 cleavage occurred in all three lines, including K562-RN although the effect of Z-vad-fmk was extremely weak in this particular cell line. This would suggest that the modifications of tyrosine kinome generated when rendering these cells resistant to TKI may be involved for a switch towards a caspase-independant apoptosis. This hypothesis is in line with the fact that tyrosine-kinase activity (several are overexpressed in K562-RN) influences DNA damage and caspase-independent apoptosis as shown in breast cancer cells [6, 27]. This could also explain that H2AX phosphorylation in response to MPA is lower in K562-RN cells compared to the other two lines which do not overexpress tyrosine-kinases. A caspase-independent cell death has been reported in Bcr-Abl-positive cells treated by a combination of TKI and Z-Vad-fmk which harbour a necrosis-like phenotype [28]. In their model, Okada et al. show the role of the serine protease Omi/HtrA2 in a necrosis-like death. This death could be involved in the response of K562 cells to MPA. However, incubation with the serine protease inhibitor ucf-101, which inhibits Omi/HtrA2, did not modify MPA-induced cell death suggesting again a different death than necrosis (data not shown).

In addition to the weak apoptotic response, we have observed a large and significant senescent-like cell cycle arrest in MPA-treated K562 cells associated to a large increase in SA-*β*-galactosidase-positive cells. This would suggest that MPA induced senescence in K562 cells. Indeed, stress induced senescence can be triggered by p53-dependent pathways and p16/INK4 upregulation. However, these pathways cannot be activated in our cells in which these genes are deleted. Senescence has been shown to be triggered by p53- and p16- independent pathway that could be indirectly through cell cycle arrest and ATM activation [29, 30]. Indeed, the senescent response is not the behavior of K562 cells alone but also occurred in lama-84 and AR-230 cells and it was in a similar proportion than apoptosis. In contrast, some other cell lines respond to MPA by apoptosis only like KCL22 or Baf3 cells expressing Bcr-Abl (Drullion, unpublished results).

MPA is able to block proliferation and to induce death of primary CD34 CML cells either sensitive or resistant to imatinib or nilotinib. Although CD34 CML cells respond to MPA mainly by apoptosis, it may be a powerful tool in the evolved CML disease or TKI resistant like those harbouring the T315I mutation or overexpressing tyrosine kinases. This also suggests that K562 cells possess additional molecular anomalies that rely on the blast phase. Despite an arrest of proliferation, we were not able to detect SA-*β*-Gal in MPA-treated CD34 CML cells. Interestingly, RNA silencing of p53 in other CML cell line did not switch MPA- induced apoptosis to a senescent response confirming that other candidates play roles (Drullion, unpublished results).

Since autophagy has been shown to induce cell surviving by the rescue of the cells from apoptosis or by cell death depending on the cellular context, we thought of interest to study this response in MPA-treated K562 cells. The increased expression of LC3B indicates that MPA induced autophagy certainly through the inhibition of the mTor pathway as suggested by the study of Gu et al. [13]. To the best of our knowledge, this is the first report showing that MPA is able to induce autophagy in CML cells. Autophagy has been shown to rescue CML cells from TKI-induced apoptosis [31–33]. Here, we see that the similar thing happens in MPA-treated cells and that inhibition of autophagy increases apoptosis in the K562- treated cells. This emphasizes that MPA- induced apoptosis is weak partially because the cells are rescued by autophagy. Regarding senescence, however, we failed to observe any effect of autophagy inhibition. The inhibition of autophagy by anti-ATG7 shRNA did not abolish or modify MPA-induced senescent-like cell cycle arrest. In light of what was described about the positive role of autophagy on stress-induced senescence, different mechanisms may explain the absence of regulation of MPA-induced senescence by autophagy in our model [34]. Even no significant inhibitory role of autophagy has been pointed out, the senescent response did not require an upstream autophagic response to occur as the inhibition of the later did not abolish senescence. It will be interesting to check in our model if the lack of a positive regulation of senescence by autophagy is correlated to the absence of expression of the different autophagic-related genes discovered to be up-regulated during senescence [34]. Another explanation may be that MPA- induced autophagy by noncanonical pathway like it was described for resveratrol [35, 36]. This peculiar pathway triggering a noncanoical autophagy is described independent of Beclin 1 and of inhibition of Vps34, a PI3 kinase, by 3 MA. In our hands, 3-MA was inhibiting MPA-induced autophagy leading to an increase of cell mortality mainly by apoptosis but not by senescence. The inhibition of the expression of ATG7 in K562 cells decreases autophagy although it failed to fully block the transformation of LC3BI in LC3BII suggesting that residual autophagy can occur. This could explain why we were unable to demonstrate a link between autophagy and senescence in response to MPA. Moreover, the inhibition of autophagy by 3 MA or siRNA against LC3 did not change the senescence response to MPA arguing for an absence of role. These results also highlight that the relations between the different deaths observed in a specific model are not a constant rule. We were not able to demonstrate an inhibition or conversely potentiating of MPA-induced senescent-like cell cycle arrest whatever the way used to block autophagy. Indeed, this suggests that in contrast to apoptosis, autophagy is not able to rescue MPA-treated cells from senescence.

One interesting point to induce a senescent-like cell cycle arrest or apoptosis in CML disease is that resistance to TKI-treatment and persistence of leukemic cells are associated to relapse and the evolution of the disease. MPA is able to target both, CML cells of the chronic phase, like we did with CML primary cells, and blastic cells, like we did with CML cell lines. Such molecule is actively looked for. In conclusion, molecules triggering cell death independently of tyrosine kinase inhibition are interesting tools to get rid of resistant CML cells and may be one way to check in blast cells from acute leukemia. MPA which triggers apoptosis and senescent-like cell cycle arrest is one of these interesting drugs.

## Supplementary Material

Figure 1: Inhibition of K562 cell proliferation by MPA K562-S, K562-R and K562-RN cells (2.10^5^/ml) (2.10^5^/ml) were grown in the presence of vehicle only, MPA (3*μ*g/ml) or incubated with guanosine (200 *μ*M) and MPA (3 *μ*g/ml) for 3 days. At day 3, cell proliferation was measured by counting cells using trypan blue exclusion assay. Cell viability from triplicate counting is expressed as the mean of 7 independent experiments (A).
Figure 2: MPA induced nuclear foci in K562 cells K562 cells were grown in the presence of vehicle only or MPA (3 *μ*g/ml) for 3 days. K562 cells (5.10^4^) were fixed in PFA and then permeabilized with triton X-100 (0.1%) for 5 min at room temperature. After one wash in PBS, slides were incubated in the presence of Dapi (1 *μ*g/ml) for 5 min at room temperature. After washes, cells were visualized under an inverted microscope. Pictures were acquired and analyzed using the NIS Nikon software.
Figure 3: MPA-induced autophagy is limiting death K562-S, K562-R and K562-RN cells were grown in the presence of vehicle only, MPA (3 *μ*g/ml) or incubated with 3-methyl adenine (3-MA, 2.5 mM), chloroquine (CQ, 25 *μ*M) and MPA (3 *μ*g/ml) for 3 days. K562 cells (5.10^4^) were incubated for 15 min in the presence of annexin-V and propidium iodide. Samples were analyzed for annexin-V and PI positive cells by flow cytometry. Results show the % of annexin V-labelled cells (A, *n* = 5). K562 cells (10^5^) were fixed in PFA and then incubated overnight in a 96 wells plate in the presence of X-Gal (1mg/ml) at 37°C as described in Figure 3. SA-*β*-gal positive cells were quantified by counting 10^2^ cells on three separate fields for each condition. Results show the mean of six independent experiments. (B, *n*= 6).
Figure 4: Inhibition of CD34 cell proliferation by MPA Primary CD34 cells isolated from blood samples of CML patients responding to imatinib were grown in the presence of vehicle only, imatinib 1 *μ*M or MPA 3 *μ*g/ml for 3 days. Cell morphology was observed upon treatment at day 3 (A) or used for detection of SA-*β*-gal positive cells (B).
Figure 5: CD34 cells of TKI sensitive CML patient respond to imatinib by apoptosis but addition of MPA did not increase the apoptotic response (A). In contrast, CD34 cells from TKI- resistant patient did not respond to imatinib as suspected but achieved apoptosis in response to MPA. The response of CD34 CML cells to MPA is partially reverted by guanosine addition (B) even for the highestMPA concentration required to reach the optimal apoptotic response C).
