# Supplementary material for: Mycophenolic Acid Overcomes Imatinib and Nilotinib Resistance of Chronic Myeloid Leukemia Cells by Apoptosis or a Senescent-Like Cell Cycle Arrest
Source: Leuk Res Treatment. 2012 Feb 23;2012:861301. doi: 10.1155/2012/861301 (PMC3504262; doi:10.1155/2012/861301)
Supplement: Supplementary file 1 — Figure 1: Inhibition of K562 cell proliferation by MPA K562-S, K562-R and K562-RN cells (2.105/ml) (2.105/ml) were grown in the presence of vehicle only, MPA (3μg/ml) or incubated with guanosine (200 μM) and MPA (3 μg/ml) for 3 days. At day 3, cell proliferation was measured by counting cells using trypan blue exclusion assay. Cell viability from triplicate counting is expressed as the mean of 7 independent experiments (A). Figure 2: MPA induced nuclear foci in K562 cells K562 cells were grown in the presence of vehicle only or MPA (3 μg/ml) for 3 days. K562 cells (5.104) were fixed in PFA and then permeabilized with triton X-100 (0.1%) for 5 min at room temperature. After one wash in PBS, slides were incubated in the presence of Dapi (1 μg/ml) for 5 min at room temperature. After washes, cells were visualized under an inverted microscope. Pictures were acquired and analyzed using the NIS Nikon software. Figure 3: MPA-induced autophagy is limiting death K562-S, K562-R and K562-RN cells were grown in the presence of vehicle only, MPA (3 μg/ml) or incubated with 3-methyl adenine (3-MA, 2.5 mM), chloroquine (CQ, 25 μM) and MPA (3 μg/ml) for 3 days. K562 cells (5.104) were incubated for 15 min in the presence of annexin-V and propidium iodide. Samples were analyzed for annexin-V and PI positive cells by flow cytometry. Results show the % of annexin V-labelled cells (A, n = 5). K562 cells (105) were fixed in PFA and then incubated overnight in a 96 wells plate in the presence of X-Gal (1mg/ml) at 37°C as described in Figure 3. SA-β-gal positive cells were quantified by counting 102 cells on three separate fields for each condition. Results show the mean of six independent experiments. (B, n= 6). Figure 4: Inhibition of CD34 cell proliferation by MPA Primary CD34 cells isolated from blood samples of CML patients responding to imatinib were grown in the presence of vehicle only, imatinib 1 μM or MPA 3 μg/ml for 3 days. Cell morphology was observed upon treatment at day 3 ( [file 861301.f1.pdf]

Supplementary Figure 1

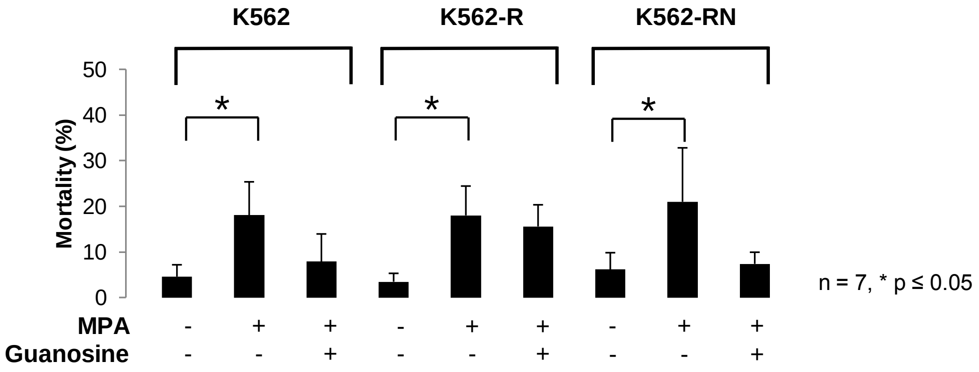

Drullion et al,.

Supplementary Figure 2

K562-S cells

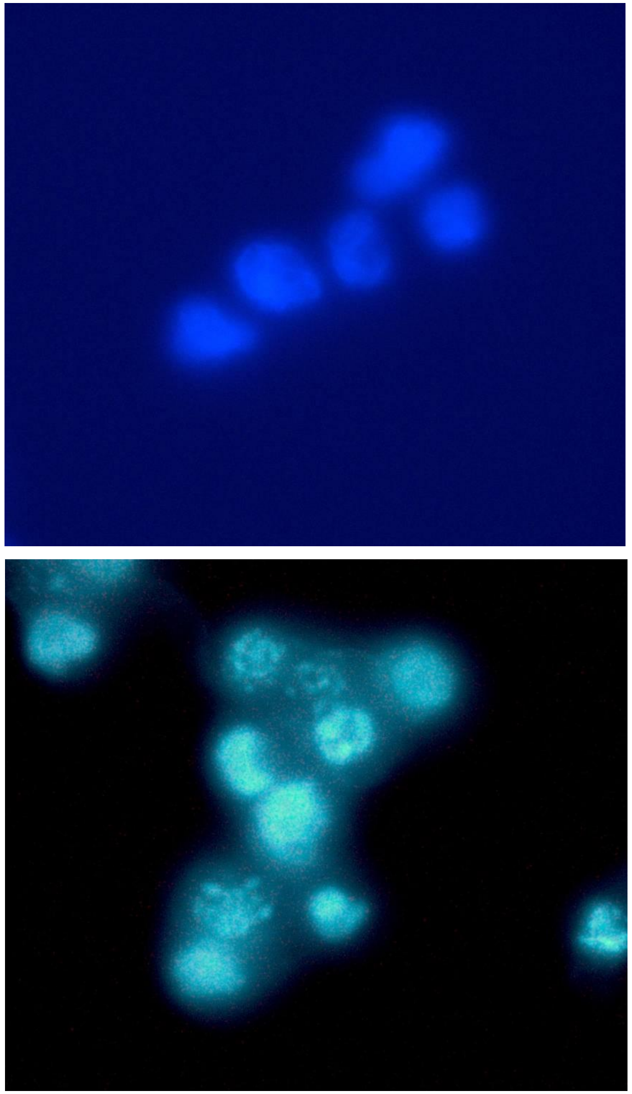

Control

MPA
3 µg/ml 72h

Drullion et al,.

Supplementary Figure 3

A

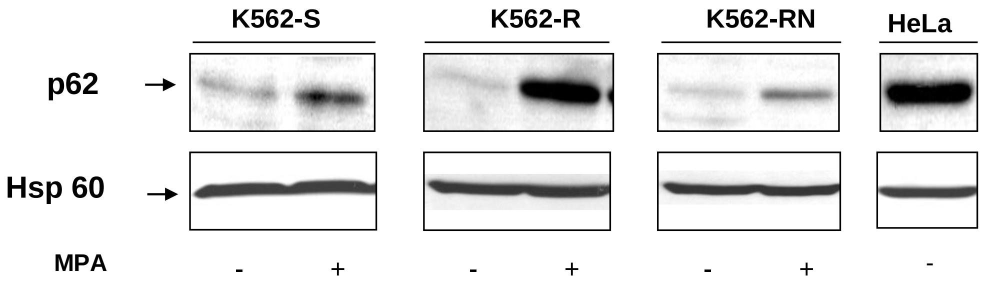

Drullion et al,.

Supplementary Figure 3

B

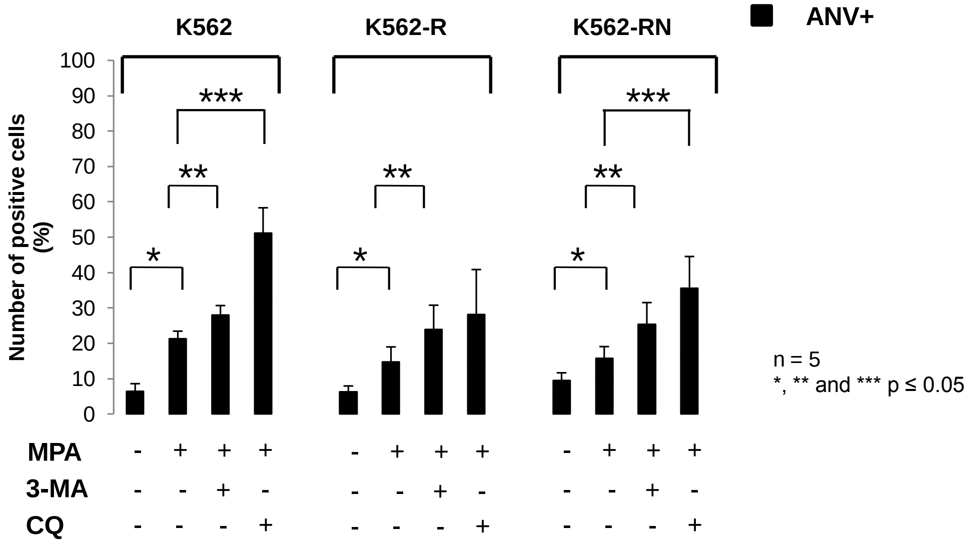

C

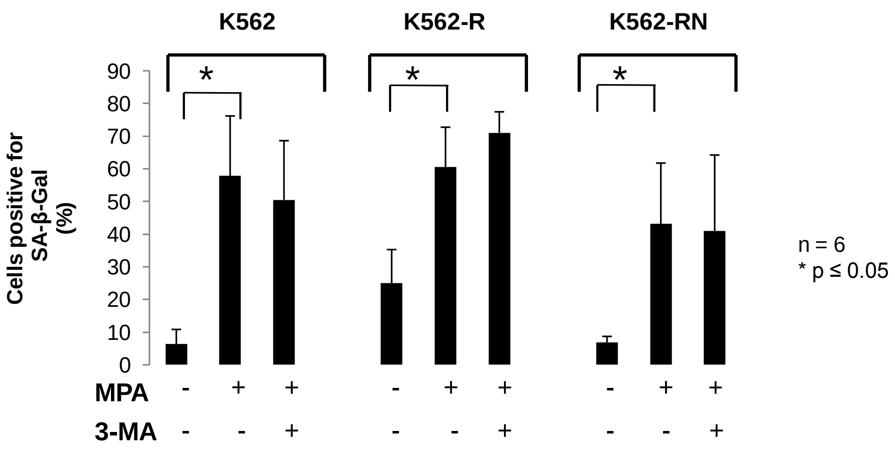

Drullion et al,.

Supplementary Figure 3

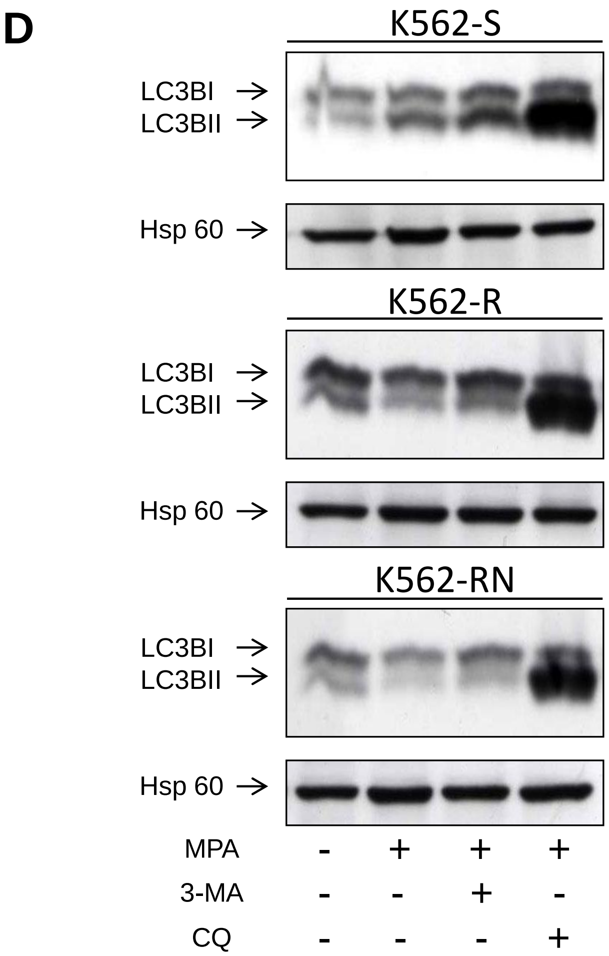

Drullion et al,.

Supplementary Figure 4

CD34+ positive cells

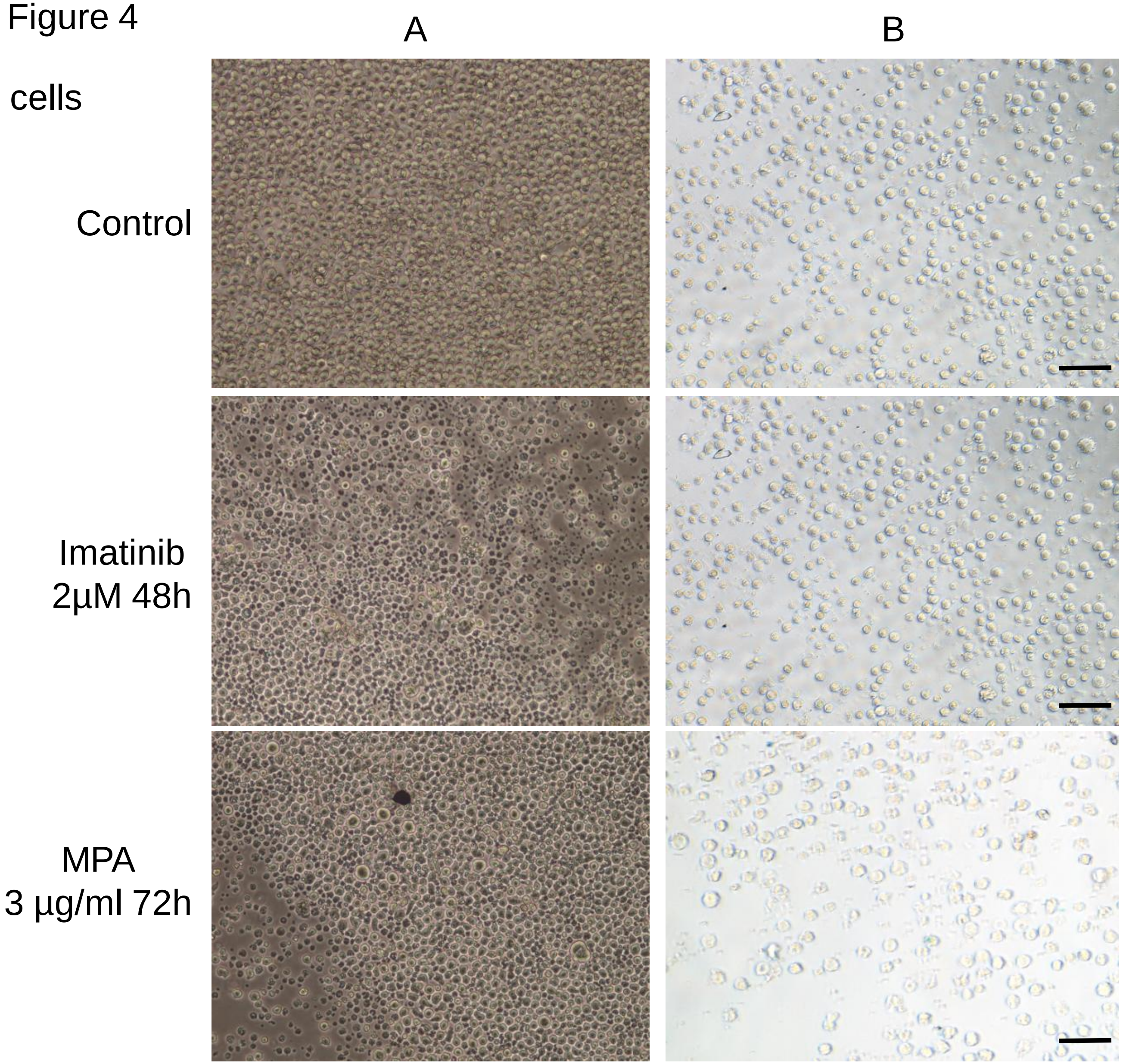

Supplementary Figure 5

A

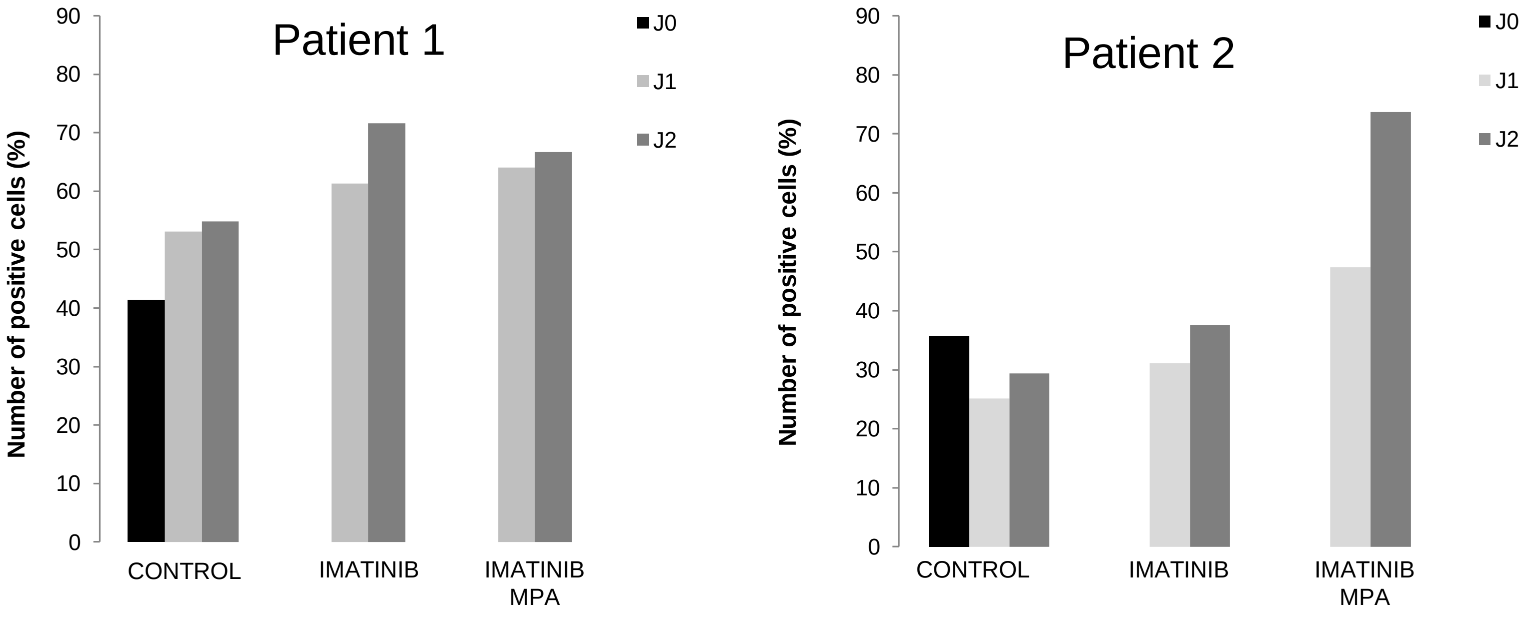

Drullion et al,.

Supplementary Figure 5

B

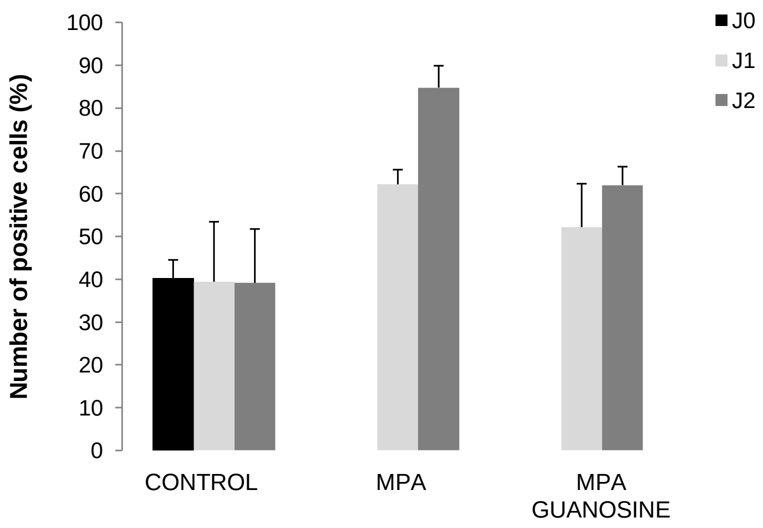

Drullion et al,.

Supplementary Figure 5

C

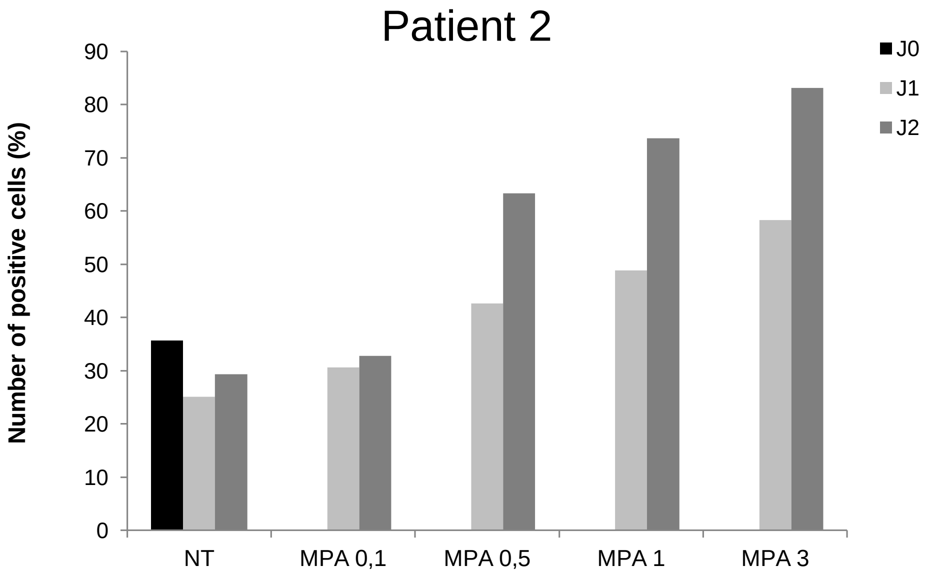

Drullion et al,.
